# Infectious Clones Produce SARS-CoV-2 That Causes Severe Pulmonary Disease in Infected K18-Human ACE2 Mice

**DOI:** 10.1128/mBio.00819-21

**Published:** 2021-04-20

**Authors:** Xiang Liu, Ali Zaid, Joseph R. Freitas, Nigel A. McMillan, Suresh Mahalingam, Adam Taylor

**Affiliations:** a Menzies Health Institute Queensland, Griffith University, Gold Coast Campus, Southport, Queensland, Australia; b Global Virus Network (GVN) Centre of Excellence in Arboviruses, Griffith University, Gold Coast, QLD, Australia; c School of Medical Sciences, Griffith University, Gold Coast, QLD, Australia; Johns Hopkins Bloomberg School of Public Health

**Keywords:** coronavirus, cytokines, infectious clones, lung infection, respiratory viruses

## Abstract

Newly emerged severe acute respiratory syndrome coronavirus 2 (SARS-CoV-2) is the causative agent of the ongoing coronavirus disease 2019 (COVID-19) pandemic, which has caused extensive mortality and morbidity and wreaked havoc on socioeconomic structures. The urgent need to better understand SARS-CoV-2 biology and enable continued development of effective countermeasures is aided by the production of laboratory tools that facilitate SARS-CoV-2 research. We previously created a directly accessible SARS-CoV-2 toolkit containing user-friendly reverse genetic (RG) infectious clones of SARS-CoV-2. Here, using K18-human ACE2 (hACE2) mice, we confirmed the validity of RG-rescued SARS-CoV-2 viruses to reproduce the infection profile, clinical disease, and pathogenesis already established in mice infected with natural SARS-CoV-2 isolates, often patient derived. RG-rescued SARS-CoV-2-infected K18-hACE2 mice developed substantial clinical disease and weight loss by day 6 postinfection. RG-rescued SARS-CoV-2 was recovered from the lungs and brains of infected K18-hACE2 mice, and infection resulted in viral pneumonia with considerable changes in lung pathology, as seen previously with natural SARS-CoV-2 infection. In mice infected with RG-rescued SARS-CoV-2-mCherry, mCherry was detected in areas of lung consolidation and colocalized with clinically relevant SARS-CoV-2-assocated immunopathology. RG-rescued SARS-CoV-2 viruses successfully recapitulated many of the features of severe COVID-19 associated with the K18-hACE2 model of SARS-CoV-2 infection. With utility *in vivo*, the RG-rescued SARS-CoV-2 viruses will be valuable resources to advance numerous areas of SARS-CoV-2 basic research and COVID-19 vaccine development.

## Abstract (precis)

To develop COVID-19 countermeasures, powerful research tools are essential. We produced a SARS-COV-2 reverse genetic (RG) infectious clone toolkit that will benefit a variety of investigations.

## INTRODUCTION

Coronavirus disease 2019 (COVID-19) caused by severe acute respiratory syndrome coronavirus 2 (SARS-COV-2) emerged from Wuhan, China, in late 2019 and rapidly developed into a global pandemic. As of 14 February 2021, the World Health Organization has reported over 108 million confirmed clinical cases of COVID-19 and over 2.3 million deaths worldwide (https://www.who.int/emergencies/diseases/novel-coronavirus-2019/situation-reports). Notably, the emergence and rapid spread of new pathogenic variants of SARS-COV-2 (1–5) and the recent resurgence of COVID-19 in Brazil (6) are making this global health crisis more intractable. There is therefore urgent global demand for tools and resources to investigate numerous aspects of SARS-COV-2 biology. Among these tools, an infectious cDNA (icDNA) clone system for generating live SARS-CoV-2 is an essential element to facilitate investigations.

A number of SARS-COV-2 reverse genetic (RG) systems based on the yeast artificial chromosome (YAC) or bacterial artificial chromosome (BAC) have been used to generate live virus following either *in vitro* or *in vivo* transcription (7–9). We recently developed and characterized a user-friendly RG infectious clone (pCC1-4K-SARS-CoV-2-Wuhan-Hu1, GenBank accession no. MT926410) from which the original SARS-CoV-2-Wuhan-Hu-1 virus (Wuhan-Hu-1) can be propagated (10). In addition, three recombinant SARS-CoV-2 infectious clones expressing various reporter cassettes, including mCherry, ZsGreen, and Nanoluciferase, were generated based on the original infectious clone. These reporter viruses provide versatility to the researcher’s toolkit and are directly accessible online (10; https://mrcppu-covid.bio/). The infectious clones will be of benefit to a variety of studies, including viral functional genomics, protein spatiotemporal dynamics, and virus-host interactions.

Small-animal models of SARS-CoV-2 infection that accurately replicate human disease have provided fundamental insights into the pathogenesis of COVID-19 (11–14). These animal models, which have been developed largely using SARS-CoV-2 isolated from patient samples, are vital to the development of countermeasures, such as vaccines and antivirals, to prevent and treat COVID-19 (15). To demonstrate the utility of our RG-rescued SARS-CoV-2 viruses in recapitulating SARS-CoV-2 replication and dissemination *in vivo* and the immunopathogenesis associated with infection, we used the human angiotensin-converting enzyme 2 (hACE2)-K18 transgenic mouse model, which expresses hACE2 driven by the keratin 18 (K18) promoter. Other mouse models of SARS-CoV-2 infection, including transient expression of hACE2 using adenovirus vectors, are unable to replicate severe disease (16). hACE2-K18 mice have been shown to develop severe lung inflammation and impaired function upon SARS-CoV-2 infection (11). Here, we characterized the infection with, and immune response to, our RG-SARS-CoV-2 and SARS-CoV-2-mCherry infectious clones using this transgenic mouse model. We report that using our RG-SARS-CoV-2 infectious clones, infection of hACE2-K18 mice reproduces the clinical signs and pathology associated with severe COVID-19, validating the use of these critical resources by the research community. These infectious clones will be valuable tools for future studies of SARS-CoV-2 biology, vaccine, and therapeutic development against COVID-19.

## RESULTS

### K18-hACE2 mice infected with RG-rescued SARS-CoV-2 isolates present with clinical disease and weight loss.

**FIG 1:**
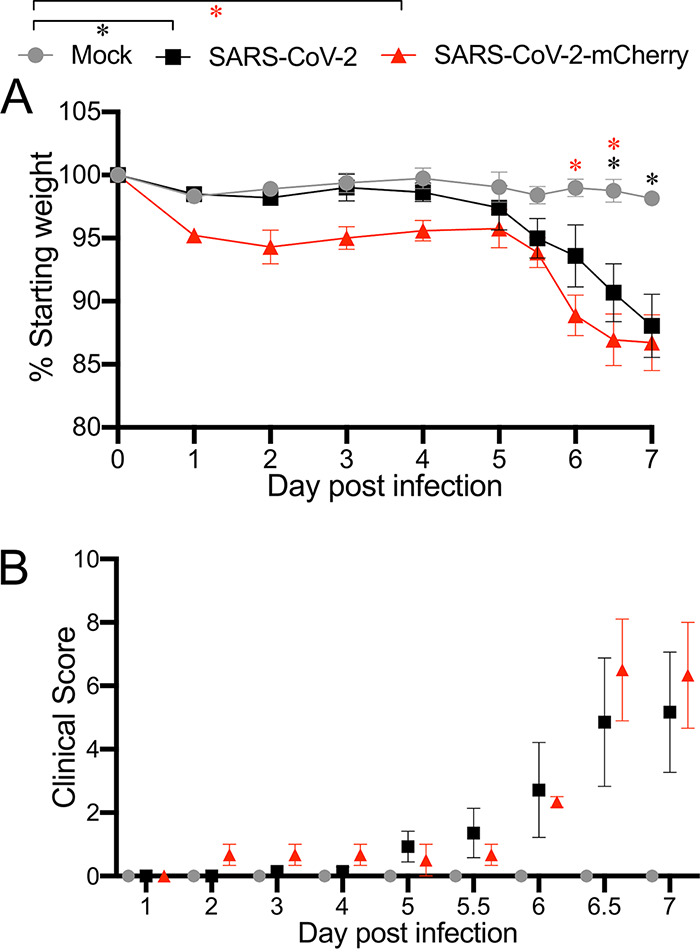
Weight loss and clinical disease score of K18-hACE2 mice infected with SARS-CoV-2 or SARS-CoV-2-mCherry. Twenty-one-week-old male and female K18-hACE2 mice were intranasally inoculated with 4 × 10^4^ PFU of SARS-CoV-2 (*n *=* *7) or SARS-CoV-2-mCherry (*n *=* *3) (20 μl per mouse). Mock-infected mice (*n *=* *5) received 20 μl sterile DMEM with 2% FBS intranasally. Individual mice were monitored daily until reaching a clinical score of >3, when twice daily monitoring was performed. (A) Weight change was monitored and compared with the initial weight on day 0. *, *P < *0.05 (two-way ANOVA with Bonferroni posttest). (B) Mice were given a clinical score according to general health (eating habit, locomotion, behavior), appearance, and weight loss. All values represent the means ± standard errors of the mean from one experiment.

Previous studies have demonstrated that hACE2-K18 mice were susceptible to infection with SARS-COV-2 isolated from patient samples and developed severe lung pathology (11–13). To determine whether virus derived from our RG SARS-CoV-2 infectious clones was pathogenic, 21-week-old male and female K18-hACE2 mice were intranasally inoculated with 4 × 10^4^ plaque-forming units (PFU) of SARS-CoV-2 (*n *=* *7, 5 male and 2 female) or SARS-CoV-2-mCherry (*n *=* *3, 2 male and 1 female). Mock-infected mice (*n *=* *5, 3 male and 2 female) received 20 μl sterile Dulbecco’s modified Eagle’s medium (DMEM) with 2% fetal bovine serum (FBS) intranasally. Beginning at day 6 postinfection, both SARS-CoV-2- and SARS-CoV-2-mCherry-infected mice showed significant weight loss compared to mock-infected mice (Fig. 1A). By day 7 postinfection, SARS-CoV-2- and SARS-CoV-2-mCherry-infected mice had lost over 10% of their starting body weight (Fig. 1A). Clinical manifestations were also recorded (Fig. 1B). Some variability in the clinical signs of SARS-CoV-2 and SARS-CoV-2-mCherry infection was observed, with clinical scores ranging from 0 to 13 on day 7 postinfection. One SARS-CoV-2-infected mouse was euthanized on day 6.5 due to the severity of disease signs, including severely restricted movement, labored breathing, and dry eyes. Despite low mouse numbers for comparison, no sex-based differences in disease were observed. Mock-infected mice did not exhibit any clinical symptoms or weight loss.

### Virus replication and tissue tropism of RG-rescued SARS-CoV-2 strains in infected K18-hACE2 mice.

**FIG 2:**
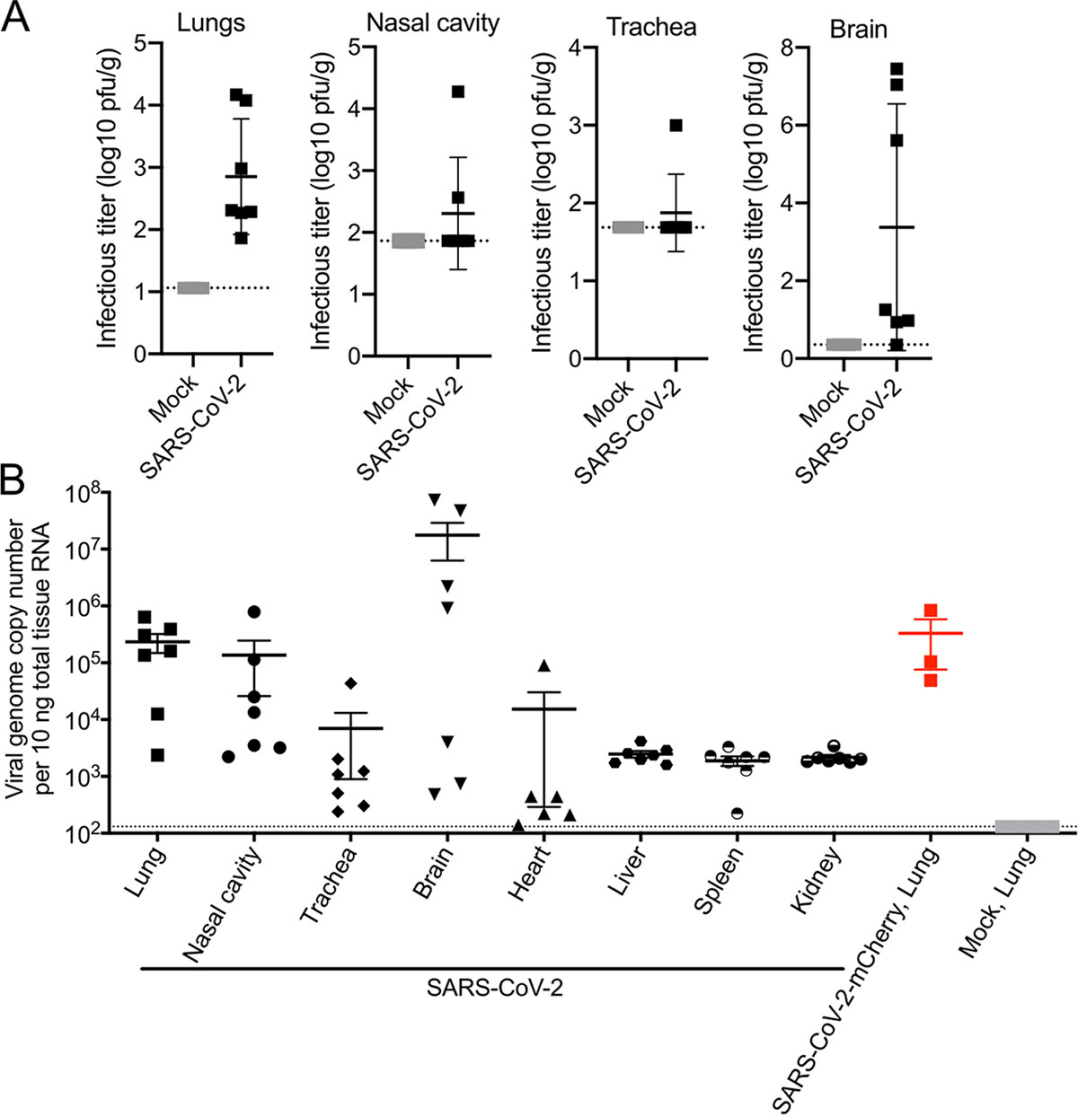
Viral burden in tissues. Twenty-one-week-old male and female K18-hACE2-transgenic mice were inoculated intranasally with 4 × 10^4^ PFU SARS-CoV-2 or SARS-CoV-2-mCherry. (A and B) At day 7 postinfection, we determined the titer of infectious virus in the lung, nasal cavity, trachea, and brain by plaque assay (A), and the viral genome copy number in tissues (lung, nasal cavity, trachea, brain, heart, liver, spleen, and kidney) was measured by RT-qPCR (B). Each symbol represents the mean titer for one mouse. Bars represent means ± standard errors, and a horizontal dotted line indicates the limit of detection.

All mice were euthanized on day 7 postinfection to assess viral loads and virus dissemination. High levels of infectious virus were detected in lung tissues of SARS-CoV-2-infected mice by plaque assay (Fig. 2A). Infectious virus was detected in the nasal cavities of two (out of seven) SARS-CoV-2-infected mice and the trachea of one mouse (out of seven) (Fig. 2A). No infectious virus was detected in the heart (data not shown). Interestingly, as shown previously, a subset of infected K18-hACE2 mice had high levels of viral RNA and infectious virus in the brain (Fig. 2A and B) (11, 17). High levels of viral RNA were detected in the lungs, nasal cavity, and, in a proportion of mice, brain tissue, consistent with the detection of infectious virus (Fig. 2B). Lower levels of viral RNA were detected in the trachea, heart, liver, spleen, and kidney (Fig. 2B). SARS-CoV-2-mCherry-infected mice had a level of viral RNA in the lung tissue similar to that of SARS-CoV-2-infected mice at day 7 postinfection: on average, 3 × 10^5^ genome copy numbers per 10 ng total RNA.

### Histological analysis of K18-hACE2 mice infected with RG-rescued SARS-CoV-2 viruses.

**FIG 3:**
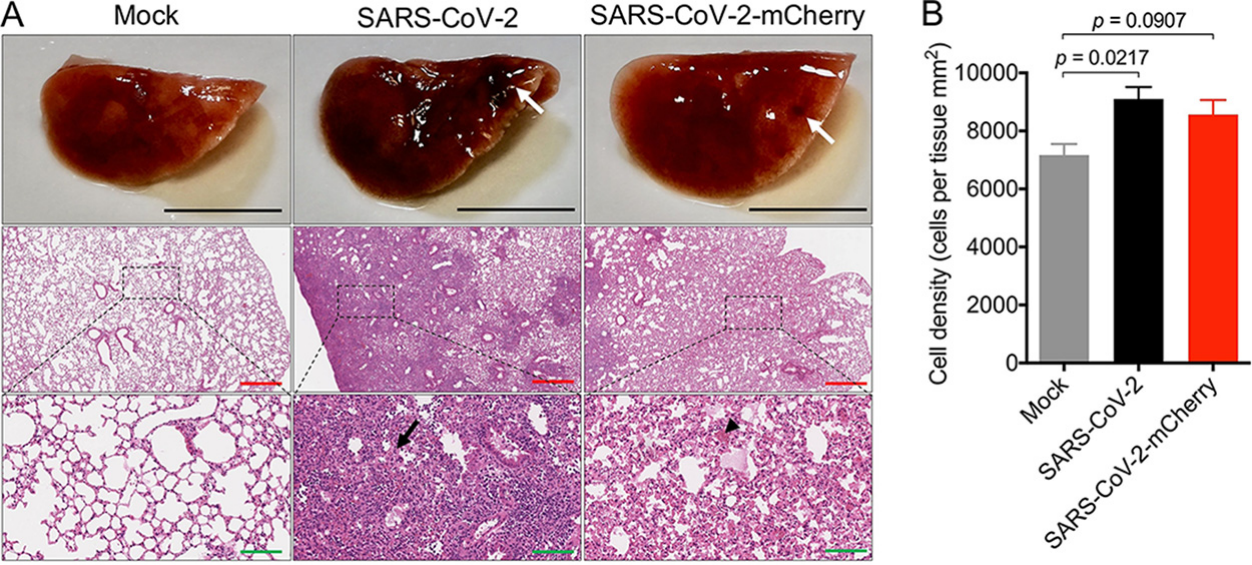
Histopathological analysis of lung tissue from SARS-CoV-2- and SARS-CoV-2-mCherry-infected K18-hACE2 mice. Twenty-one-week-old male and female K18-hACE2-transgenic mice were inoculated intranasally with 4 × 10^4^ PFU SARS-CoV-2 or SARS-CoV-2-mCherry. (A) At day 7 postinfection, lung tissue was harvested and stored in 4% PFA for 24 h prior to macroimaging. White arrows show the dark-red lesions throughout the dorsal region of the lung in infected mice. Hematoxylin and eosin (H&E)-stained lung sections demonstrate thickened interalveolar septa (black arrow) and a hemorrhage (black arrowhead) in SARS-CoV-2- and SARS-CoV-2-mCherry-infected mice. Scale bars, 6 mm (black), 500 μm (red), 100 μm (green). Black dashed boxes indicate the area of magnification. Each image is representative of ≥3 mice. (B) Cell density was quantified using ImageScope software. Statistical analyses were performed using an unpaired *t* test. Data represent means ± standard errors.

At day 7 postinfection, the left lung was harvested for histological analysis. Dissection of the lungs of SARS-CoV-2- and SARS-CoV-2-mCherry-infected mice revealed a mottled appearance that was not observed in mock-infected mice. Lungs were fixed in 4% PFA, and their gross pathology was examined (Fig. 3A). Focal dark-red lesions were observed on the surface of SARS-CoV-2- and SARS-CoV-2-mCherry-infected lung tissue (Fig. 3A), as reported previously in mice infected with SARS-CoV-2 isolated from a patient (13). Lung sections were stained with hematoxylin and eosin (H&E) to examine pathology and quantify leukocyte infiltration. Areas of the lung in SARS-CoV-2- and SARS-CoV-2-mCherry-infected mice were characterized by thickened interalveolar septa, immune cell infiltration, and signs of edema and hemorrhage (Fig. 3A and B). The focal dark-red lesions observed at the lung surface upon dissection in SARS-CoV-2- and SARS-CoV-2-mCherry-infected mice are likely due to hemorrhage and inflammation, as indicated by the histopathological profile of the infected lung (Fig. 3A).

### Inflammatory gene expression in K18-hACE2 mice infected with RG-rescued SARS-CoV-2 viruses.

**FIG 4:**
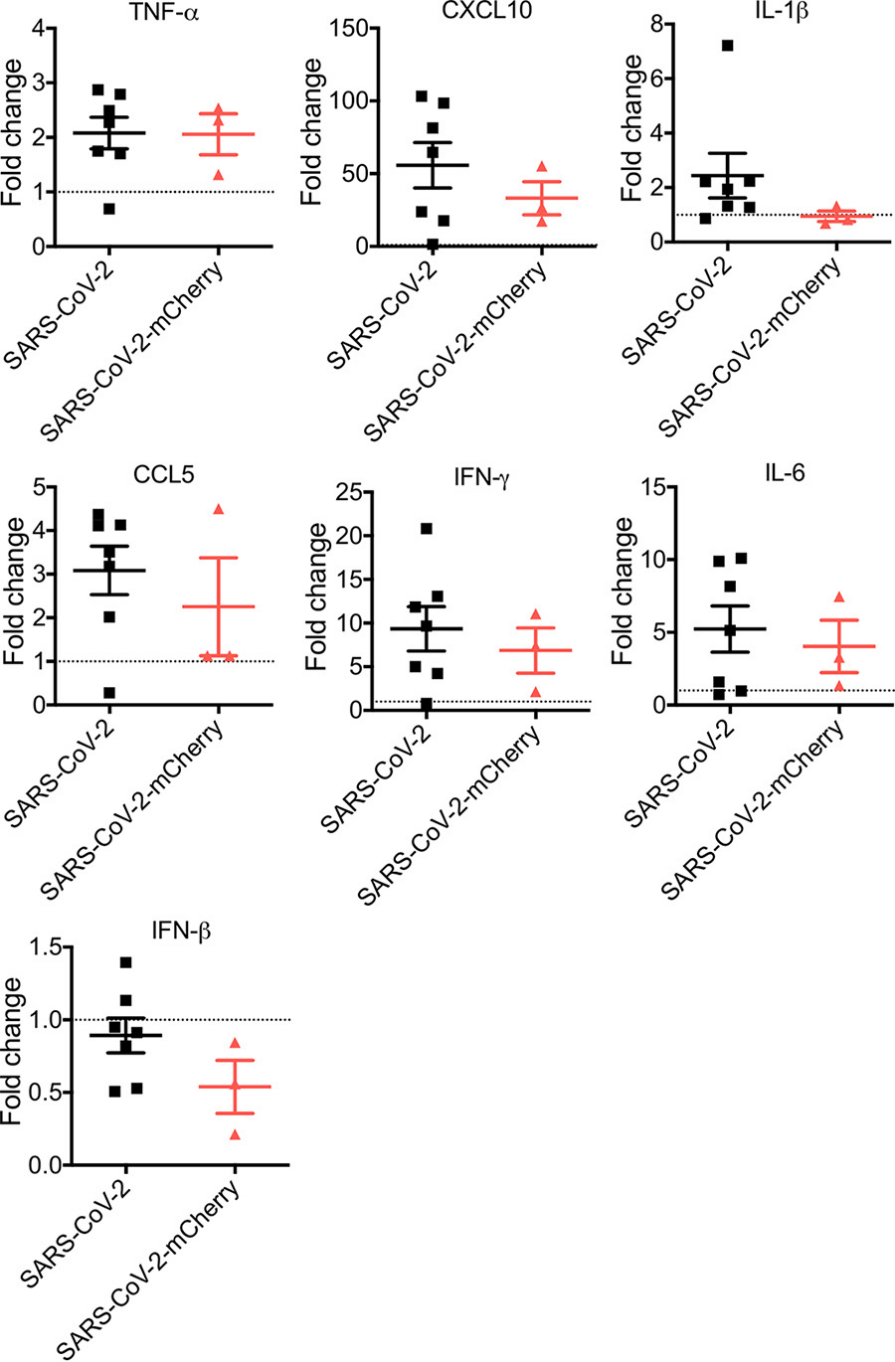
Cytokine and chemokine expression in the lung tissue of SARS-CoV-2-infected K18-hACE2 mice. Twenty-one-week-old male and female K18-hACE2-transgenic mice were inoculated intranasally with 4 × 10^4^ PFU SARS-CoV-2 or SARS-CoV-2-mCherry. Transcriptional profiles of immune mediators, namely, TNF-α, CXCL10, IL-1β, CCL5, IFN-γ, IL-6, and IFN-β, were determined by qRT-PCR in the lung at day 7 postinfection. Data were normalized to GAPDH levels and are shown as fold change from the level in mock-infected mice. Data are presented as means ± standard errors. No statistical difference in the levels of expression of inflammatory mediators was observed between SARS-CoV-2- and SARS-CoV-2-mCherry-infected lungs by the Student unpaired *t* test.

Dysregulation of inflammatory mediators in the lungs of SARS-CoV-2-infected mice is consistent with the cytokine storm seen in severe clinical cases of COVID-19 (11, 18). At day 7 postinfection, compared to those in mock-infected mice, the levels of expression of tumor necrosis factor alpha (TNF-α), CXCL10, CCL5, gamma interferon (IFN-γ), and interleukin 6 (IL-6) were upregulated in lung homogenates of SARS-CoV-2- and SARS-CoV-2-mCherry-infected mice (Fig. 4). As seen previously, baseline IFN-β mRNA expression was observed in SARS-CoV-2- and SARS-CoV-2-mCherry-infected mice at day 7 postinfection (Fig. 4) (11).

### Localization of infection in the lung tissue of SARS-CoV-2-mCherry-infected mice.

**FIG 5:**
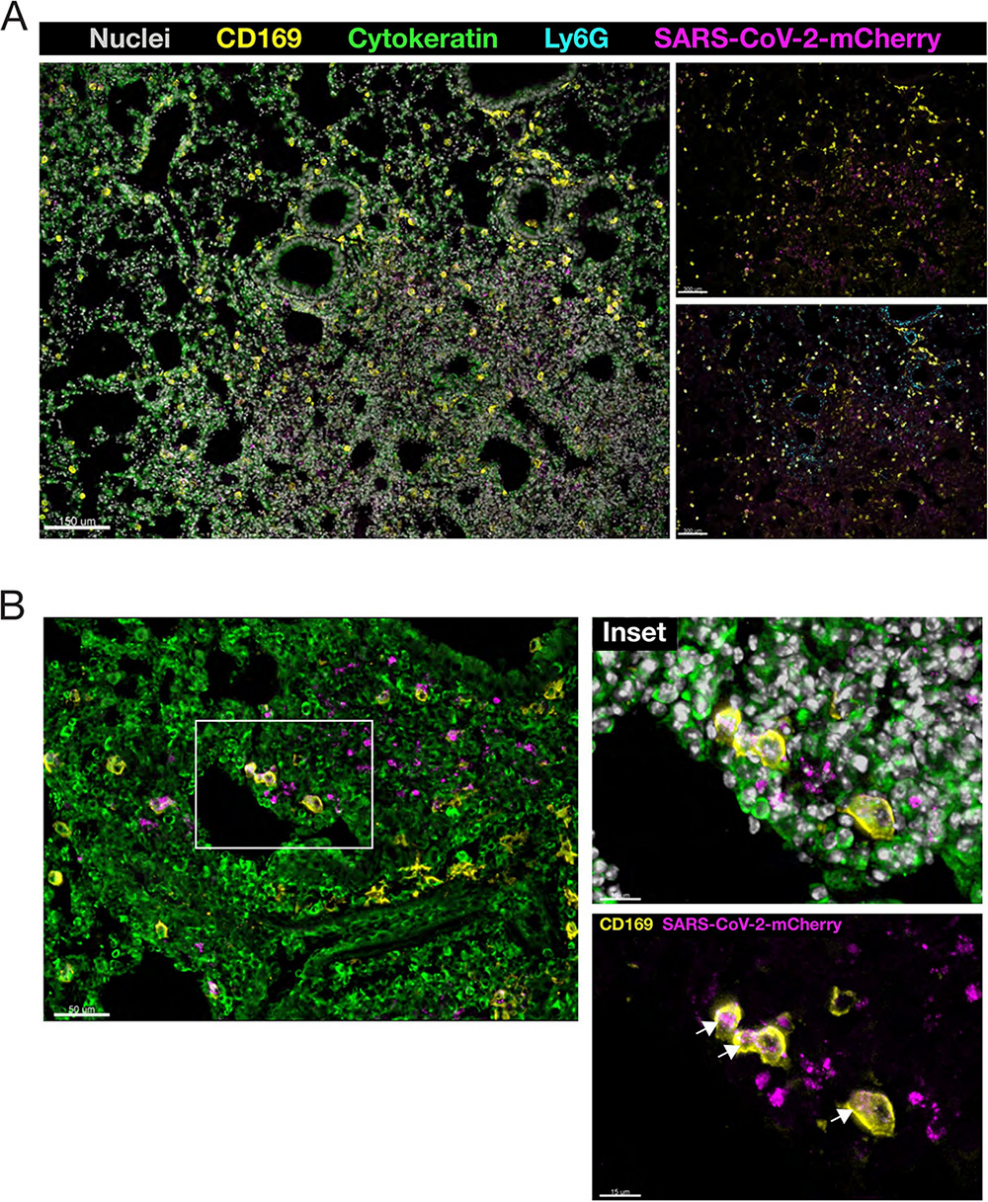
Immunofluorescence analysis of lung sections from SARS-CoV-2-mCherry-infected K18-hACE2 mice. Lung cryosections were labeled with Hoechst 33258 dye (nuclei), CD169 (alveolar macrophages), Ly6G (neutrophils), and cytokeratin 14 (parenchymal tissue), and images were acquired by confocal microscopy. (A) Localization of SARS-CoV-2-mCherry in the lungs of infected mice at 7 days postinfection. The image was acquired as a z-stack using a 10× (0.75-NA) objective. (B) Colocalization of CD169^+^ alveolar macrophages and mCherry^+^ signal in the lung parenchyma. Arrows show cytoplasmic colocalization (insets). The image was acquired as a z-stack using a 20× (0.95-NA) objective.

Having detected high levels of infectious virus and viral RNA in the lungs of infected mice, we performed immunofluorescence analysis of lung sections to localize mCherry reporter expression *in situ* using confocal microscopy. Reporter mCherry signal was readily detectable in the lung tissue (Fig. 5A) and was localized primarily in highly consolidated areas of the lung. Interestingly, SARS-CoV-2-mCherry^+^ cells were confined to the parenchyma, and little or no signal was detected on the airway epithelium. Using immunolabeling, we detected CD169^+^ alveolar macrophages (AMs) and Ly6G^+^ neutrophils in the lungs of infected mice. AMs have been shown to play a critical role in the initiation of a cytokine storm in severe COVID-19, and reports have shown that they are susceptible to infection with SARS-CoV-2 (19–21). Neutrophils, which have also been shown to play a role in lung inflammation during SARS-CoV-2 infection through the release of neutrophil extracellular traps (NETs) were detected in the parenchyma, although in lower numbers, and were more sparsely distributed than AMs (22, 23). Of note, CD169^+^ AMs were found in the vicinity of, and colocalizing with, the mCherry^+^ signal (Fig. 5B, inset) in the parenchyma, corroborating reports that this subset of lung-resident macrophages actively responds to infection in the lung and is likely to be susceptible to SARS-CoV-2.

### Detection of SARS-CoV-2-mCherry in tissues of infected K18-hACE2 mice.

**FIG 6:**
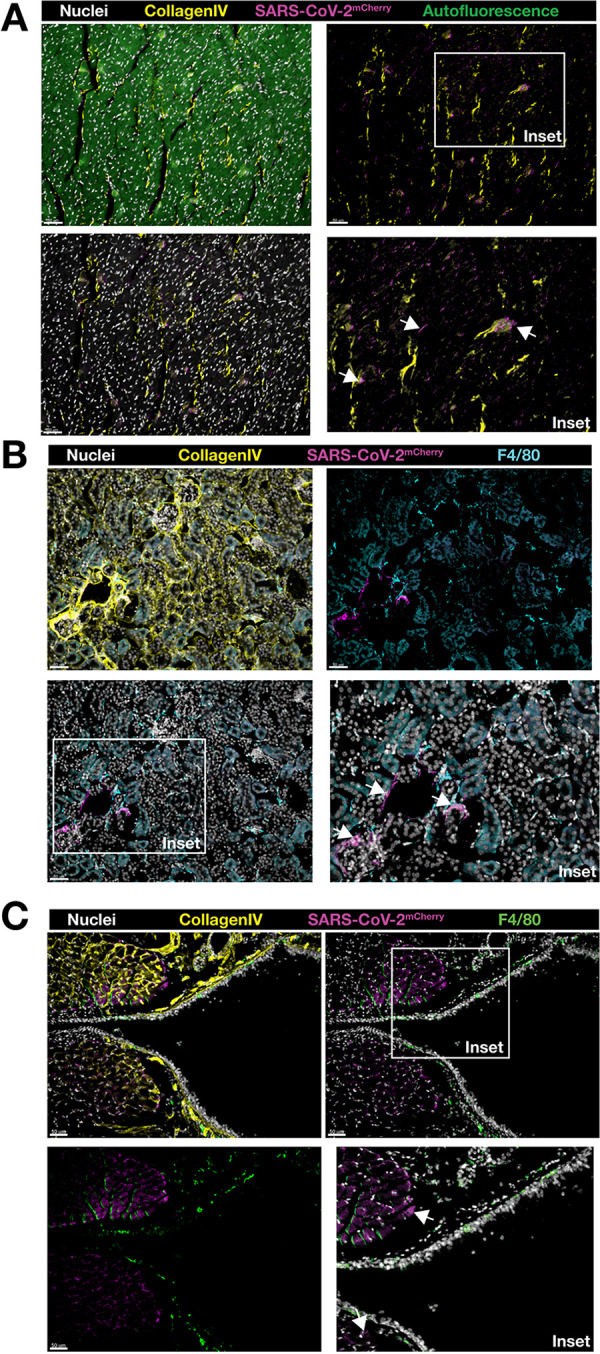
Immunofluorescence analysis of heart, kidney, and trachea sections from SARS-CoV-2-mCherry-infected K18-hACE2 mice. Tissue cryosections were labeled with Hoechst 33258 dye (nuclei), collagen type IV (basement membrane), and F4/80 (macrophage), and images were acquired by confocal microscopy. Localization of SARS-CoV-2-mCherry in the heart (cross-section) (A), kidney (cross-section) (B), and trachea (longitudinal section) (C) of infected mice at 7 days postinfection. Images were acquired as a z-stack using a 20× (0.95-NA) objective. Arrows show mCherry-positive cells (insets). Scale bars (50 μm) are shown in individual panels.

To investigate the dissemination of SARS-CoV-2-mCherry infection in K18-hACE2 mice, we performed immunofluorescence analysis of heart (Fig. 6A), kidney (Fig. 6B), and trachea (Fig. 6C) tissue to localize mCherry reporter expression *in situ* using confocal microscopy. Reporter mCherry signal was readily detectable in the heart and trachea (Fig. 6A and C), with foci of SARS-CoV-2-mCherry^+^ cells detected in the kidney (Fig. 6B).

## DISCUSSION

The majority of SARS-CoV-2 infections in humans are mild or subclinical (24). Symptoms of COVID-19 can range from fever, cough, and fatigue to severe respiratory illness and death (25). Patient comorbidities increase the risk of hospitalization and severe COVID-19 (26, 27). Using virus isolated from patients, the K18-hACE2 mouse model of SARS-CoV-2 infection has been shown to reproduce the features of severe COVID-19 (11). Weight loss and clinical disease together with high viral load in the lungs and lung pathology make the K18-hACE2 mouse model of SARS-CoV-2 infection a valuable platform for vaccine and therapeutic testing.

We confirmed that the RG-rescued SARS-CoV-2 viruses exhibited replication capabilities, virulence, and pathogenicity similar to those of natural SARS-CoV-2 isolates in K18-hACE2 mice. Mice infected with RG-rescued SARS-CoV-2 viruses showed significant weight loss and clinical disease by day 6 postinfection. We observed variability in the clinical scores between infected mice as seen previously in K18-hACE2 mice infected with patient-isolated SARS-CoV-2 (12). Variability in clinical scores may be due to inconsistencies in the intranasal route of inoculation and subsequent distribution to the lung. Robust replication of RG-rescued SARS-CoV-2 viruses in the lungs was accompanied with detection of live virus in the trachea and nasal cavity as well as the brain, hallmarks of natural SARS-CoV-2 infection in previous studies (17). Tissues positive for SARS-CoV-2 infection have previously been shown to productively express the hACE2 transgene in K18-hACE2 mice (11). One caveat to using the K18-hACE2 model of disease is the inconsistency in hACE2 expression between humans and mice, particularly in the brain (11). Our studies accurately replicated the stratified titers of both viral RNA and live virus recovered from the brains of infected K18-hACE2 mice at day 7 postinfection (11). These results further clarify the utility of RG-rescued SARS-CoV-2 viruses to replicate natural SARS-CoV-2 infection in K18-hACE2 mice.

Histopathological analysis showed clear pulmonary pathological changes in infected mice, including cellular infiltration, consolidation, and thickened interalveolar septa. Elevated production of inflammatory mediators at day 7 postinfection, a late time point, indicates that a profound immune response resulted from infection and aligns with increased levels of inflammatory mediators, including IL-1β, IFN-γ, TNF-α, and CXCL10, detected in COVID-19 patients (25). Sustained expression of inflammatory mediators has been reported in the lung tissue of SARS-CoV-2-infected K18-hACE2 mice (11). No change in IFN-β expression in the lung tissues of infected mice replicates the kinetics of expression seen at this late time point in previous studies (11). Immunofluorescence analysis of SARS-CoV-2-mCherry-infected lung, heart, kidney, and trachea sections confirmed the expression of reporter mCherry signal *in vivo*. mCherry signal in the lung was detected in areas of consolidation and was shown to colocalize with innate immune cells, which have been described as critical components of SARS-CoV-2 immunopathology (21, 23).

In summary, we have developed user-friendly RG-rescued SARS-CoV-2 viruses capable of expressing fluorescent reporters *in vivo* and used this system to recapitulate the pathogenesis of SARS-CoV-2 infection in the K18-hACE2 mouse model of severe COVID-19. RG-SARS-CoV-2 infectious clones are a vital tool for the research community to further dissect the biology of SARS-CoV-2. With the advantage of producing a predominantly homogenous population of RG-rescued SARS-CoV-2 viruses, the infectious clones can be used to study the intrahost evolution of SARS-CoV-2. Modification of these tools or the infectious clone strategy can be applied to study the transmissibility and virulence of emerging SARS-CoV-2 variants. Our validation that the RG-rescued SARS-CoV-2 viruses can be reliably used to model severe SARS-CoV-2 infection *in vivo* makes them valuable assets in the development of vaccines, antivirals, and therapeutics against COVID-19.

## MATERIALS AND METHODS

### DNA-launched SARS-CoV-2 plasmid construction and rescue of infectious virus.

pCC1-4K-SARS-CoV-2-Wuhan-Hu1 (GenBank accession no. MT926410) and pCC1-4K-SARS-CoV-2-mCherry (GenBank accession no. MT926411) infectious clones were assembled from synthetic DNA fragments (GenScript) using restriction endonuclease-based cloning procedures as described in detail previously (10). Briefly, 10 μg of infectious clone DNA was transfected into BHK-21 cells using Lipofectamine LTX with Plus reagent (Life Technologies) according to the manufacturer’s instructions in a T25 flask. At 24 h posttransfection, the BHK-21 cells were resuspended gently using a cell scraper, and the whole-cell slurry was transferred on to a monolayer of Vero E6 cells in a T25 flasks. Cell supernatant was collected as virus P0 stock at 48 to 72 h posttransference or until 50% cytopathic effect (CPE) was observed.

### Plaque assay.

Infectious titers were enumerated by plaque assay (28). Mouse tissues were collected in phosphate-buffered saline (PBS) and homogenized using the Bead Ruptor 24 Elite homogenizer according to the manufacturer’s instructions. The supernatant was collected by centrifugation and stored at −80°C. Vero E6 cells were seeded in 12-well plates at 2.5 × 10^5^ cells per well and cultured in DMEM with 5% FCS overnight. The cells were infected with a dilution series of virus from samples and overlaid with 1.2% colloidal microcrystalline cellulose (Sigma-Aldrich) in DMEM with 2% FCS. The plates were incubated for 72 h. Cells were fixed with 4% paraformaldehyde (PFA) and stained with 0.1% crystal violet. Viral titers were calculated using the following formula: PFU/ml = (average number of plaques/volume [ml] of virus added) × the dilution factor.

### Mouse infection and ethics statement.

All animal experiments were approved by the Animal Ethics Committee of Griffith University (protocol no. MHIQ/07/20/AEC). All procedures conformed to the *Australian Code for the Care and Use of Animals for Scientific Purposes* (29). K18-hACE2 C57BL/6 mice were obtained from the Animal Resource Centre (Perth, Australia). Twenty-one-week-old male and female K18-hACE2 mice were inoculated intranasally with 4 × 10^4^ PFU of SARS-CoV-2 (Wuhan-Hu-1) or SARS-CoV-2-mCherry derived from infectious clones in a volume of 20 μl. Mock-infected mice received 20 μl of sterile DMEM with 2% FBS. Mice were weighed and scored for disease signs daily. Mice were scored using a cumulative and progressive clinical disease matrix. Mice were given a score between 0 and 3 for each of the following five health indicators: eating habit, locomotion, behavior, appearance, and weight loss. A score of 0 was normal. After receiving an overall score of >3, mice were monitored twice daily. A score of 3 in any one of the 5 categories (e.g., >15% weight loss) or an overall score of >10 led to the mouse being euthanized.

### Immunofluorescence and confocal microscopy.

Tissues were fixed in 4% paraformaldehyde overnight at 4°C. Fixed tissues were subsequently washed in PBS and dehydrated in 30% (wt/vol) sucrose (in PBS). Tissues were embedded in optimal-cutting-temperature (OCT) compound (Sakura Finetek) and frozen prior to cryosectioning. Eighteen to twenty-micrometer-thick sections were prepared and permeabilized in cold acetone. Sections were blocked (Dako Serum-Free Protein Block; Dako) and immunolabeled with rat anti-mouse CD169 (AbD Serotec), rabbit anti-mouse cytokeratin 14 (Abcam), Alexa Fluor 647-conjugated rat anti-mouse Ly6G (BD Biosciences) antibodies, rabbit anti-mouse collagen type IV (Abcam), or rat monoclonal F4/80 (Abcam), and secondary antibody detection was performed using anti-rat Alexa Fluor 488 and anti-rabbit Alexa Fluor 546 antibodies (Thermo Fisher). Sections were washed and nuclei were labeled using Hoechst 33258 dye and mounted using ProLong Gold antifade (Thermo Fisher). Images were acquired using a spectral laser scanning confocal microscope (Olympus FV3000) using a 10× (0.75-numerical-aperture [NA]) or a 20× (0.95-NA) objective. Sequential acquisition was performed using single 405-nm, 488-nm, 561-nm, 594-nm, and 640-nm laser lines to excite Hoechst 33258 dye, Alexa Fluor 488, Alexa Fluor 546, mCherry, and Alexa Fluor 647. z-stacks with a 1-μm interval were acquired, channels were merged using Olympus CellSens software, and images were processed using Imaris (v9.5; Bitplane). Spectral overlap was resolved using the channel arithmetics function of the Imaris XT plugin, and the noniterative deconvolution algorithm was applied to z-stack images. Images were exported as 300-dpi TIFF files and assembled using Adobe Illustrator 2020.

### Histology.

Tissues were dissected and fixed in 4% PFA, followed by paraffin embedding. Samples were cut into 5-μm-thick sections and stained with hematoxylin and eosin (H&E). Images were taken using an Aperio AT2 digital whole-slide scanner (Leica). Tissue cellular infiltrates were quantified using ImageScope software (Algorithm Nuclear v9) using the following threshold settings: image zoom of 0.5, a minimum nuclear size (μm^2^) of 10, a maximum nuclear size (μm^2^) of 100, a minimum roundness of 0.4, a minimum compactness of 0.4, and a minimum elongation of 0.2.

### Real-time quantitative PCR (RT-qPCR).

Tissues were collected in TRIzol (Invitrogen) and homogenized using the Bead Ruptor 24 Elite homogenizer according to manufacturer’s instructions. Total RNA was extracted from tissue homogenates using TRIzol (Invitrogen) according to the manufacturer’s instructions. RNA was reverse transcribed into cDNA using reverse transcriptase (Sigma-Aldrich) with random primers. SYBR green and QuantiTect probe real-time PCR was performed on a CFX96 touch real-time PCR system (Bio-Rad) to measure the expression of inflammatory mediators and viral genome copy numbers, respectively. Primers for GAPDH (glyceraldehyde-3-phosphate dehydrogenase; housekeeping), TNF-α, CXCL10, IL-1β, CCL5, IFN-γ, IL-6, and IFN-β were purchased from Qiagen. SYBR green real-time PCR conditions were set as follows: (i) 1 cycle of 95°C for 15 min and (ii) 40 cycles of 94°C for 15 s, followed by 55°C for 30 s and 72°C for 30 s. SARS-COV-2 RdRP gene-targeting primer and probe sequences were 5′-GTGAAATGGTCATGTGTGGCGG-3′ (forward), 5′-CAAATGTTAAAAACACTATTAGCATA-3′ (reverse), and FAM-CAGGTGGAACCTCATCAGGAGATGC-BHQ (probe sequence), where FAM is 6-carboxyfluorescein and BHQ is black hole quencher (30). The QuantiTect probe real-time PCR conditions were set as follows: (i) 1 cycle of 95°C for 15 min and (ii) 40 cycles of 94°C for 15 s followed by 60°C for 1 min. The DNA amplification specificity was evaluated by melting curve analysis. The mRNA level of each target gene was expressed as fold change from the level in mock-infected samples. Viral genome copy number was calculated against a standard curve for a viral infectious clone (GenBank accession no. MT926410).

### Statistical analysis.

All data were tested for normality using the D’Agostino-Pearson normality test prior to analysis. Data for mouse weight was analyzed using two-way analysis of variance (ANOVA) with the Bonferroni *post hoc* test. Cytokine production and infiltrated cell counts were analyzed using an unpaired *t* test. All statistical analyses were performed with GraphPad Prism 6.0 software.
